# Electrostatic Self-Assembly of CdS Quantum Dots with Co_9_S_8_ Hollow Nanotubes for Enhanced Visible Light Photocatalytic H_2_ Production

**DOI:** 10.3390/molecules29153530

**Published:** 2024-07-26

**Authors:** Yuqing Yan, Yonghui Wu, Chenggen Lu, Yu Wei, Jun Wang, Bo Weng, Wei-Ya Huang, Jia-Lin Zhang, Kai Yang, Kangqiang Lu

**Affiliations:** 1 Jiangxi Provincial Key Laboratory of Functional Crystalline Materials Chemistry, School of Chemistry and Chemical Engineering, Jiangxi University of Science and Technology, Ganzhou 341000, China; 18797912889@163.com (Y.Y.); 13207070350@163.com (Y.W.); 17779524039@163.com (C.L.); 18225906201@163.com (Y.W.); wj15270473375@126.com (J.W.); hweiya@126.com (W.-Y.H.); jialinz2007@163.com (J.-L.Z.); yangkai@jxust.edu.cn (K.Y.); 2 CAS Key Laboratory of Urban Pollutant Conversion, Institute of Urban Environment, Chinese Academy of Sciences, Xiamen 361021, China; bweng@iue.ac.cn

**Keywords:** CdS, Co_9_S_8_, quantum dot, photocatalytic H_2_ evolution, cocatalyst

## Abstract

CdS quantum dots (CdS QDs) are regarded as a promising photocatalyst due to their remarkable response to visible light and suitable placement of conduction bands and valence bands. However, the problem of photocorrosion severely restricts their application. Herein, the CdS QDs-Co_9_S_8_ hollow nanotube composite photocatalyst has been successfully prepared by loading Co_9_S_8_ nanotubes onto CdS QDs through an electrostatic self-assembly method. The experimental results show that the introduction of Co_9_S_8_ cocatalyst can form a stable structure with CdS QDs, and can effectively avoid the photocorrosion of CdS QDs. Compared with blank CdS QDs, the CdS QDs-Co_9_S_8_ composite exhibits obviously better photocatalytic hydrogen evolution performance. In particular, CdS QDs loaded with 30% Co_9_S_8_ (CdS QDs-30%Co_9_S_8_) demonstrate the best photocatalytic performance, and the H_2_ production rate reaches 9642.7 μmol·g^−1^·h^−1^, which is 60.3 times that of the blank CdS QDs. A series of characterizations confirm that the growth of CdS QDs on Co_9_S_8_ nanotubes effectively facilitates the separation and migration of photogenerated carriers, thereby improving the photocatalytic hydrogen production properties of the composite. We expect that this work will facilitate the rational design of CdS-based photocatalysts, thereby enabling the development of more low-cost, high-efficiency and high-stability composites for photocatalysis.

## 1. Introduction

With the increasingly serious environmental pollution and the increasing demand for energy, the development and utilization of sustainable clean energy to achieve green development has become a hot topic [1,2,3,4]. In recent years, photocatalytic hydrogen evolution has attracted much attention due to its advantages of zero carbon emission, high efficiency and sustainability, and is considered a promising energy conversion method [5,6,7]. Therefore, the utilization of photocatalytic technology to produce hydrogen energy represents a feasible strategy for alleviating environmental pollution and energy crises [8,9,10]. The practical application of photocatalytic hydrogen production technology is contingent upon three key factors: low cost, high efficiency and high stability [11,12]. One of the most commonly employed modification strategies to improve the photocatalytic H_2_ evolution properties of semiconductors is the introduction of precious metals (such as Au, Ag, Pd and Pt) through doping. Nevertheless, precious metals are limited and expensive. Consequently, the development of cost-effective, environmentally friendly and highly active photocatalysts represents a significant and pressing challenge [13,14].

In recent years, metal sulfides have become a research focus in the field of photocatalytic hydrogen evolution on account of their exceptional light absorption properties and unique electronic structure. Among these, CdS has been extensively studied owing to its appropriate band gap and position of energy bands [15,16]. Moreover, CdS exhibits diverse morphologies and structures, involving zero-dimensional (0D) quantum dots, one-dimensional (1D) nanorods, two-dimensional (2D) nanosheets and three-dimensional (3D) cubes [17]. CdS QDs are considered to be a promising photocatalytic material due to their small size (<10 nm), high electron mobility and abundant recoverable light [18,19]. However, the issue of easy hole oxidation decomposition (photocorrosion) severely restricts the application of CdS [20]. Among various strategies to alleviate CdS photocorrosion, the rational incorporation of a cocatalyst is an effective approach [21]. Co_9_S_8_ serves as a widely used cocatalyst with advantages such as easy availability, abundant active sites and adjustable chemical composition [22]. In particular, the hollow-structured Co_9_S_8_ possesses a large specific surface area and enhances the absorption of light by multiple reflections, which is of significant importance in improving photocatalytic properties. Additionally, the electrostatic self-assembly method is an efficient and environmentally friendly preparation method for nanoparticles, which is expected to prepare highly active photocatalysts [23].

Herein, the CdS QDs-Co_9_S_8_ composite photocatalyst is successfully prepared through electrostatic self-assembly. Compared to blank CdS QDs, the CdS QDs-Co_9_S_8_ composite demonstrates enhanced photocatalytic H_2_ production performance. Notably, the optimal CdS QDs-30%Co_9_S_8_ exhibits a photocatalytic hydrogen production rate of 9642.7 μmol·g^−1^·h^−1^, approximately 60.3 times that of blank CdS QDs. Cyclic experiments indicate that the introduction of Co_9_S_8_ cocatalyst effectively prevents photocorrosion on the surface of CdS QDs. Moreover, subsequent characterizations confirm that loading Co_9_S_8_ cocatalyst effectively promotes the separation and migration of photogenerated carriers, thereby improving the photocatalytic properties of CdS QDs. This work illustrates the significant role of Co_9_S_8_ as a cocatalyst in the field of photocatalytic H_2_ production, and is expected to provide a useful reference for the development of effective and stable photocatalysts.

## 2. Results and Discussion

**Figure 1:**
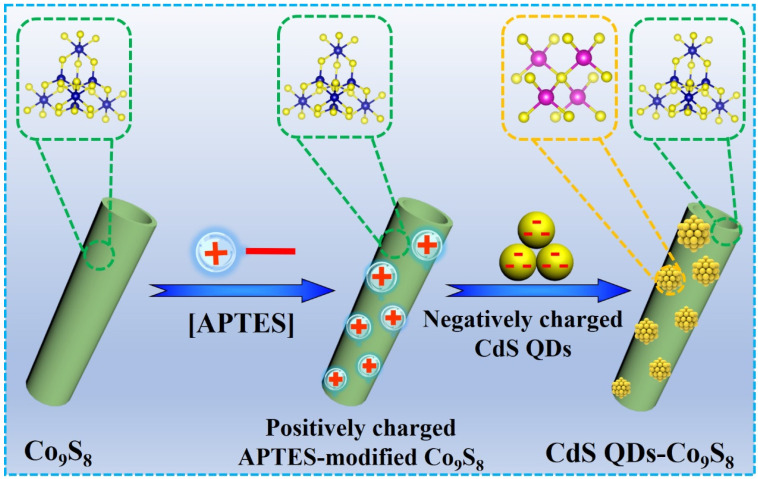
Synthesis diagram of CdS QDs-Co_9_S_8_ composite photocatalyst.

The synthesis process of the CdS QDs-Co_9_S_8_ composite photocatalyst is shown in Figure 1. Initially, the Co_9_S_8_ nanotubes are achieved through a two-step hydrothermal approach, followed by treatment with APTES to impart a positive charge. Subsequently, the treated Co_9_S_8_ nanotubes are subjected to an electrostatic assembly process with CdS QDs, resulting in the formation of the CdS QDs-Co_9_S_8_ composite photocatalyst. A diagram of the prepared samples diagram is depicted in Figure S1. As illustrated in Figure S1a,b, CdS QDs exhibit a yellow powder, while Co_9_S_8_ nanotubes display a black powder. Upon assembly of CdS QDs and Co_9_S_8_ nanotubes, the resulting CdS QDs-Co_9_S_8_ composite appears yellowish-green (Figure S1c).

**Figure 2:**
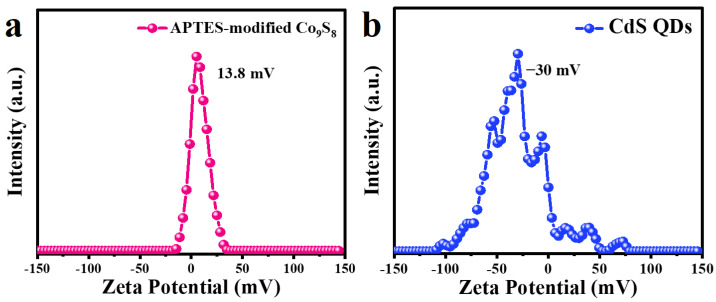
Zeta potential of (**a**) APTES-modified Co_9_S_8_ and (**b**) CdS QDs suspension dispersed in deionized water.

Figure 2a,b display the Zeta potentials of APTES-modified Co_9_S_8_ and CdS QDs suspension dispersed in deionized water, respectively. It can be observed that the Zeta potentials of APTES-modified Co_9_S_8_ and CdS QDs are 13.8 mV and −30 mV, respectively, which means that APTES-modified Co_9_S_8_ is positively charged, while CdS QDs is negatively charged. This result provides a good basis for the assembly of the CdS QDs-Co_9_S_8_ composite [24].

**Figure 3:**
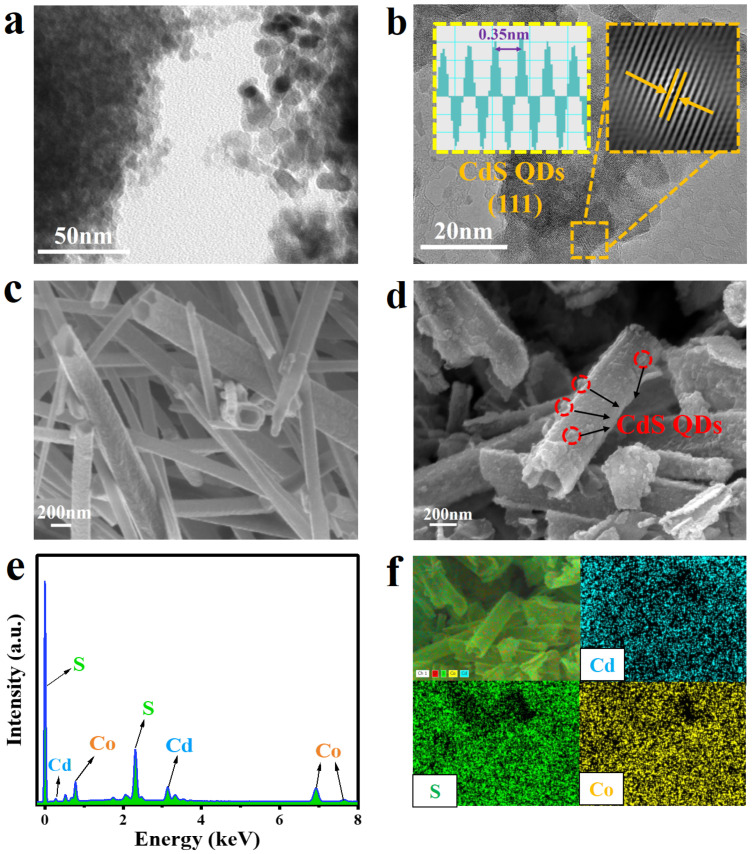
(**a**) TEM image and (**b**) HRTEM image of CdS QDs. SEM images of (**c**) Co_9_S_8_ and (**d**) CdS QDs-30%Co_9_S_8_. (**e**) The EDS spectrum of CdS QDs-30%Co_9_S_8_. (**f**) The element mapping results of CdS QDs-30%Co_9_S_8_.

Morphological and microstructural analyses of CdS QDs, Co_9_S_8_ and CdS QDs-30%Co_9_S_8_ were conducted using scanning electron microscopy (SEM) and transmission electron microscope (TEM). As shown in Figure 3a, the TEM image reveals that the diameter of CdS QDs is roughly 4 nm, consistent with previous literature [25]. Furthermore, as displayed in Figure 3b, the high-resolution TEM (HRTEM) image exhibits a lattice spacing of 0.35 nm corresponding to the (111) crystal face of CdS QDs, indicating its successful preparation [25]. Meanwhile, the TEM and HRTEM images of Co_9_S_8_ (Figure S2) demonstrate the successful synthesis of Co_9_S_8_ nanotubes. As depicted in Figure S2b, the 0.23 nm of lattice spacing corresponds to the (420) crystal face of Co_9_S_8_. Figure 3c illustrates a hollow nanotube structure with a diameter of approximately 200 nm for Co_9_S_8_. As exhibited in Figure 3d, CdS QDs-30%Co_9_S_8_ inherits the hollow nanotube structure of Co_9_S_8_. It is worth noting that the hollow structure exposes a large specific surface area and enhances the absorption of light by multiple reflections, which is of significant importance in improving the photocatalytic properties. Furthermore, it can be observed that CdS QDs are evenly decentralized on the Co_9_S_8_ nanotubes. As presented in Figure 3e, the EDS spectra illustrate the presence of Co, Cd and S elements in the CdS QDs-30%Co_9_S_8_ composite. Moreover, the composition of all the composite photocatalysts is quantitatively analyzed using inductively coupled plasma emission spectrometry (ICP-OES). As indicated in Table S1, as the Co_9_S_8_ load increases, the proportion of the Co element rises, while the proportion of the Cd element decreases, consistent with the anticipated results. In addition, the element mapping results of CdS QDs-30%Co_9_S_8_ indicate that CdS QDs are uniformly distributed on the surface of Co_9_S_8_ nanotubes (Figure 3f). These results demonstrate the successful synthesis of the CdS QDs-30%Co_9_S_8_ composite.

**Figure 4:**
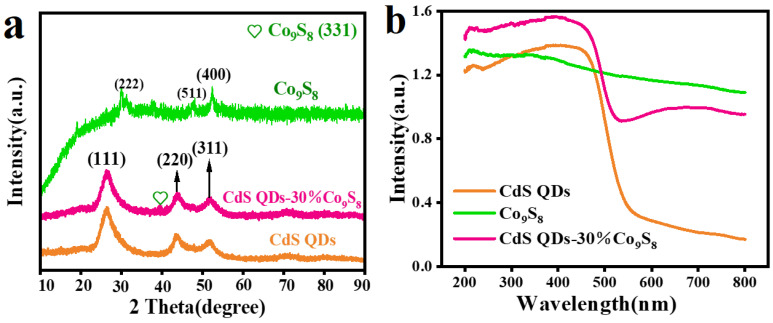
(**a**) XRD pattern of blank CdS QDs, Co_9_S_8_ and CdS QDs-30%Co_9_S_8_ composite. (**b**) UV−vis diffuse reflection spectra of blank CdS QDs, Co_9_S_8_ and CdS QDs-30%Co_9_S_8_ composite.

The crystal structure and phase composition of the prepared samples were investigated by X-ray diffraction (XRD). Figure 4a illustrates the XRD patterns of CdS QDs, Co_9_S_8_ and CdS QDs-30%Co_9_S_8_. It can be observed that the XRD peak of CdS exhibits a relatively strong intensity, indicating its robust crystal phase. In contrast, the XRD peak of Co_9_S_8_ displays a relatively weak intensity, suggesting its inferior crystal phase. For Co_9_S_8_, the diffraction peaks at 2θ = 29.9°, 31.4°, 37.4°, 39.5°, 47.5°, 52.3° and 54.6° correspond to the crystal planes (311), (222), (400), (331), (511), (400) and (531) of Co_9_S_8_, respectively (JCPDS: 65-1765) [26]. As for CdS QDs, the characteristic peaks at 26.2°, 43.6° and 51.7° can be related to the crystal faces (111), (220) and (311) of CdS (JCPDS: 75-1546), respectively [27]. In addition, the XRD diffraction curve of CdS QDs-30%Co_9_S_8_ is highly similar to that of CdS QDs, except that a weak peak at 39.5° belongs to the (331) crystal plane of Co_9_S_8_, demonstrating the successful assembly of the CdS QDs-Co_9_S_8_ composite. Furthermore, the (111) crystal face of CdS QDs exhibits a strong characteristic diffraction peak, resulting in a peak of Co_9_S_8_ at 29.9° masked by CdS QDs. The optical properties of a series of samples are determined by UV–vis diffuse reflectance spectroscopy (DRS). Figure 4b exhibits the light absorption curves of CdS QDs, Co_9_S_8_ and the CdS QDs-30%Co_9_S_8_ composite. The blank CdS presents a distinct absorption edge at near 570 nm. Moreover, Co_9_S_8_ illustrates strong absorption across the entire spectral range, suggesting excellent light collection ability from ultraviolet to visible light regions. Notably, the CdS QDs-30%Co_9_S_8_ composite displays superior light harvesting capability compared to CdS alone, which indicates the enhanced light absorption achieved through the introduction of the Co_9_S_8_ cocatalyst in the composite photocatalyst.

**Figure 5:**
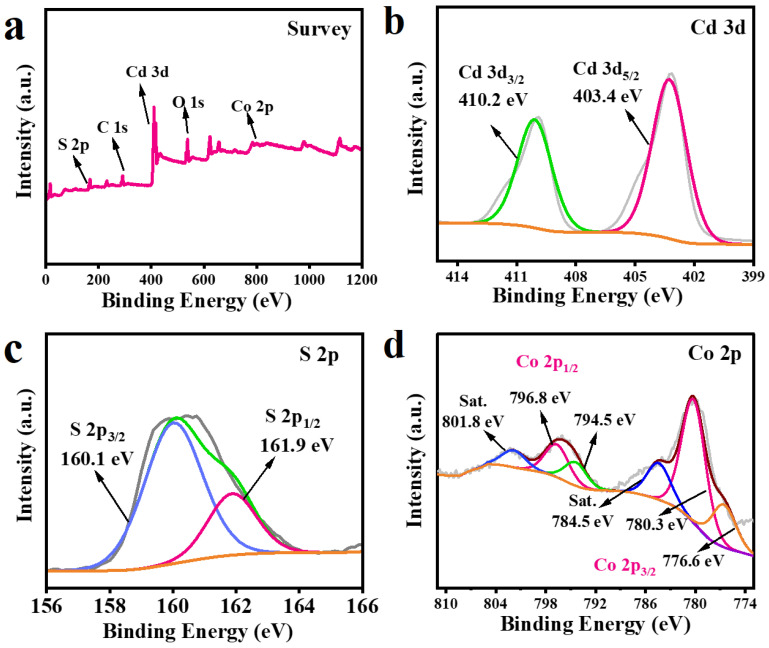
XPS spectra for (**a**) the survey spectra of the CdS QDs-30%Co_9_S_8_ composite, (**b**) Cd 3d, (**c**) S 2p and (**d**) Co 2p.

X-ray photoelectron spectroscopy (XPS) analysis (Figure 5) of the CdS QDs-30%Co_9_S_8_ composite is performed in order to further determine the chemical state and elemental composition of the prepared sample. As the survey spectra shown in Figure 5a, Co, Cd and S elements are present in the CdS QDs-30%Co_9_S_8_ composite, which further confirms the successful assembly of CdS QDs and Co_9_S_8_ cocatalyst. In the XPS spectra of Cd 3d (Figure 5b), the two characteristic peaks at 410.2 eV and 403.4 eV belong to Cd 3d_3/2_ and Cd 3d_5/2_, respectively, which demonstrates Cd exists in the form of +2 valence in the binary composite photocatalyst CdS QDs-30%Co_9_S_8_ [21]. As illustrated in Figure 5c, the distinct peaks at the binding energies of 160.1 eV and 161.9 eV belong to S 2p_3/2_ and S 2p_1/2_, respectively, confirming the existence of S^2−^ [28]. In addition, the XPS spectra of Co 2p displayed in Figure 5d can be divided into two spin-orbital dual peaks and two satellite peaks (identified as “Sat.”). The first dual peaks at 780.3 eV and 776.6 eV and the second dual peaks at 796.8 eV and 794.5 eV can be attributed to Co 2p_3/2_ and Co 2p_1/2_, respectively, demonstrating the existence of Co^2+^ and Co^3+^ [29]. The XPS results confirm that the prepared composite contains CdS and Co_9_S_8_, which indicates the successful preparation of this hybrid.

**Figure 6:**
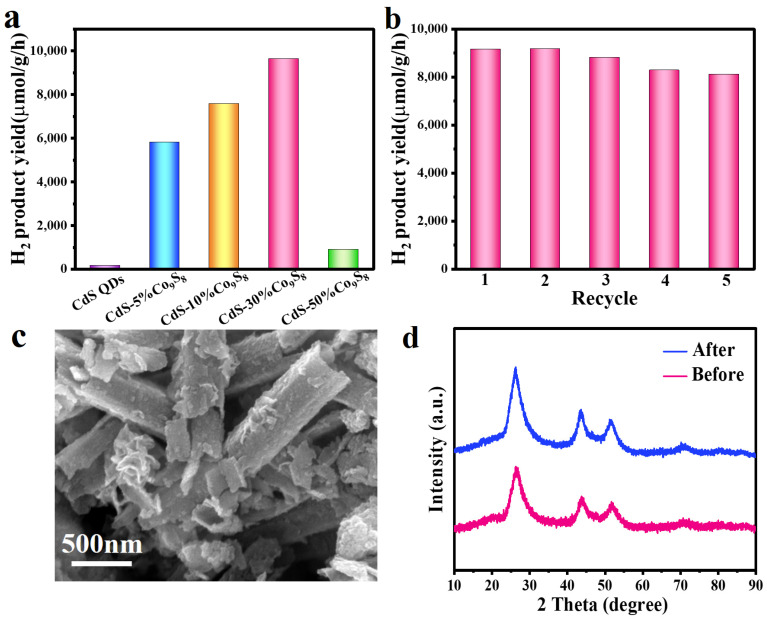
(**a**) Photocatalytic hydrogen production rates of blank CdS QDs and CdS QDs-Co_9_S_8_ composite. (**b**) Cyclic stability test of CdS QDs-30%Co_9_S_8_ photocatalytic hydrogen production. (**c**) The SEM images of CdS QDs-30%Co_9_S_8_ composite after cyclic test. (**d**) XRD patterns of the CdS QDs-30%Co_9_S_8_ before and after cyclic test.

In order to compare the photocatalytic performance of pure CdS QDs and CdS QDs-Co_9_S_8_ composites illuminated by visible light, a photocatalytic hydrogen evolution experiment is conducted using TEOA as a sacrificial agent. As displayed in Figure 6a, on account of the serious recombination of photogenerated carriers, the blank CdS QDs exhibit low photocatalytic activity, which demonstrates a hydrogen production rate of 159.8 μmol·g^−1^·h^−1^. When CdS QDs are combined with 5%, 10%, 30% and 50% Co_9_S_8_ nanotubes, the different proportions of the CdS QDs-Co_9_S_8_ composites show enhanced photocatalytic activity. As the loading capacity of Co_9_S_8_ is increased, the photocatalytic H_2_ production rate of the CdS QDs-Co_9_S_8_ composites exhibits a corresponding increase. In particular, the optimal CdS QDs-30%Co_9_S_8_ composite photocatalyst demonstrated a hydrogen production rate of 9642.7 μmol·g^−1^·h^−1^, which is 60.3 times that of pure CdS QDs. Nevertheless, when the Co_9_S_8_ cocatalyst content continually increased, the hydrogen production rate of the CdS QDs-Co_9_S_8_ composite decreased. This phenomenon may be attributed to the high proportion of cocatalysts, which results in the masking of the CdS QDs’ active sites during hydrogen evolution. As demonstrated in Table 1, the photocatalytic H_2_ evolution rate of the CdS QDs-30%Co_9_S_8_ composite is superior to that of similar photocatalysts documented in the literature. Furthermore, the photocatalytic stability of the CdS QDs-30%Co_9_S_8_ composite photocatalyst is evaluated by cyclic experiment. As illustrated in Figure 6b, the CdS QDs-30%Co_9_S_8_ composite photocatalyst demonstrates a stable photocatalytic activity following five cycles. These findings demonstrate that the CdS QDs-Co_9_S_8_ composite is an efficacious and stable photocatalyst. In addition, Figure 6c,d demonstrate that there is no obvious change in the SEM image and XRD pattern of the CdS QDs-30%Co_9_S_8_ composites following cycling, which further shows that the composites have excellent stability.

**Figure 7:**
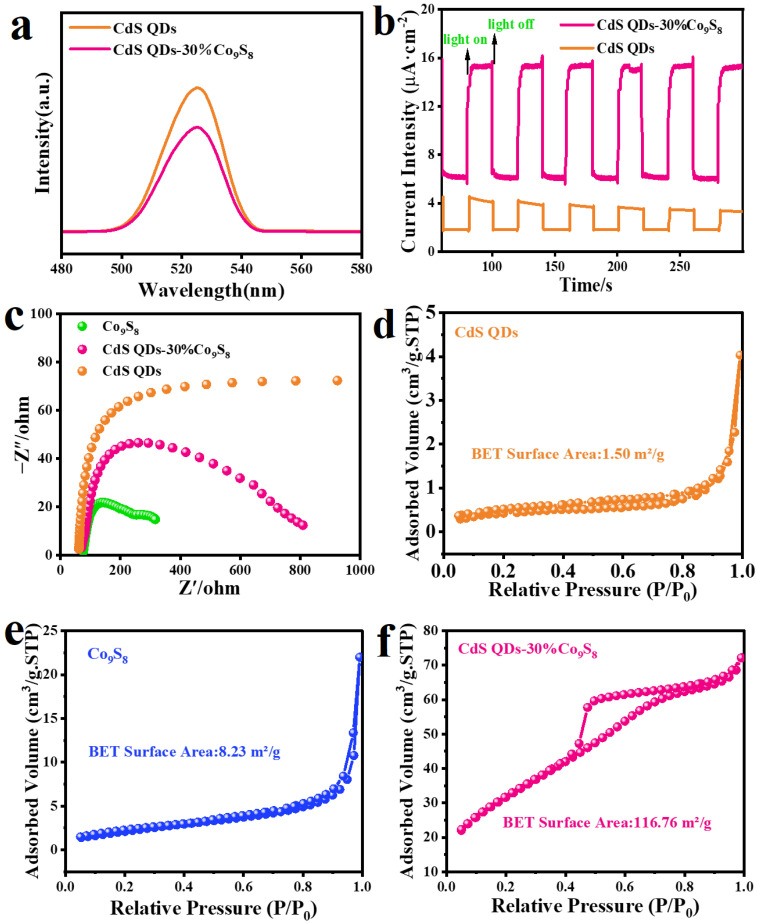
(**a**) Steady-state photoluminescence (PL) emission spectra with an excitation wavelength of 500 nm. (**b**) Transient photocurrent spectra. (**c**) EIS Nyquist plots. Nitrogen adsorption–desorption isotherms of (**d**) blank CdS QDs, (**e**) Co_9_S_8_ and (**f**) CdS QDs-30%Co_9_S_8_ composite.

The separation efficiency of photogenerated carriers can be evaluated through the photoluminescence (PL) measurement. As displayed in Figure 7a, the CdS QDs-30%Co_9_S_8_ composite photocatalyst exhibits lower PL intensity than blank CdS QDs, indicating that the introduction of the Co_9_S_8_ cocatalyst has an effective inhibition effect on the photogenerated electron–hole pair recombination, which can enhance the photocatalytic hydrogen evolution performance [39,40,41]. Figure 7b shows the instantaneous photocurrent response of blank CdS QDs and CdS QDs-30%Co_9_S_8_ [42]. As demonstrated in Figure 7b, the photocurrent response of CdS QDs-30%Co_9_S_8_ composite is significantly higher than that of blank CdS QDs, which indicates the improved photogenerated carrier separation efficiency of the CdS QDs-30%Co_9_S_8_ composite [43,44,45]. Additionally, the charge migration behavior at the catalyst–electrolyte interface is investigated through electrochemical impedance spectroscopy (EIS). Generally, a smaller radius of curvature results in lower resistance for the catalyst during the charge transfer process. As we can see from the EIS Nyquist diagram (Figure 7c), the CdS QDs-30%Co_9_S_8_ composite exhibits a smaller curvature radius than that of the CdS QDs, which demonstrates that the CdS QDs-30%Co_9_S_8_ composite represents a faster-photogenerated carrier transfer rate and a lower charge transfer resistance [46,47,48,49]. As displayed in Figure 7d,e, both CdS QDs and Co_9_S_8_ exhibit type IV isotherms in their N_2_ adsorption–desorption isotherms, indicating the mesoporous nature of these materials. The pore size distribution of CdS QDs, Co_9_S_8_ and CdS QDs-30%Co_9_S_8_ is illustrated in Figure S3 and Table S2, further confirming their mesoporous characteristics. Furthermore, the BET surface areas of CdS QDs and Co_9_S_8_ are 1.50 m^2^/g and 8.23 m^2^/g, respectively, while that of CdS QDs-30%Co_9_S_8_ is 116.76 m^2^/g (Figure 7f). The larger BET surface area of the hybrid photocatalyst compared to that of Co_9_S_8_ and CdS QDs suggests that the structure of quantum dots on hollow nanotubes can expand the catalyst’s surface area, thereby enhancing the photocatalytic properties of the composite catalysts. Moreover, the N_2_ adsorption isotherm and corresponding BET-specific surface areas of all other ratios of the composites have been investigated to identify discernible trends. As depicted in Figure S4, all composites exhibit higher BET-specific surface area than blank CdS and Co_9_S_8_, and their BET-specific surface area roughly decreases with increasing Co_9_S_8_ load. This phenomenon may be attributed to the high loading amount of Co_9_S_8_, resulting in nanotube stacking and consequently reducing the composite’s BET-specific surface area.

**Figure 8:**
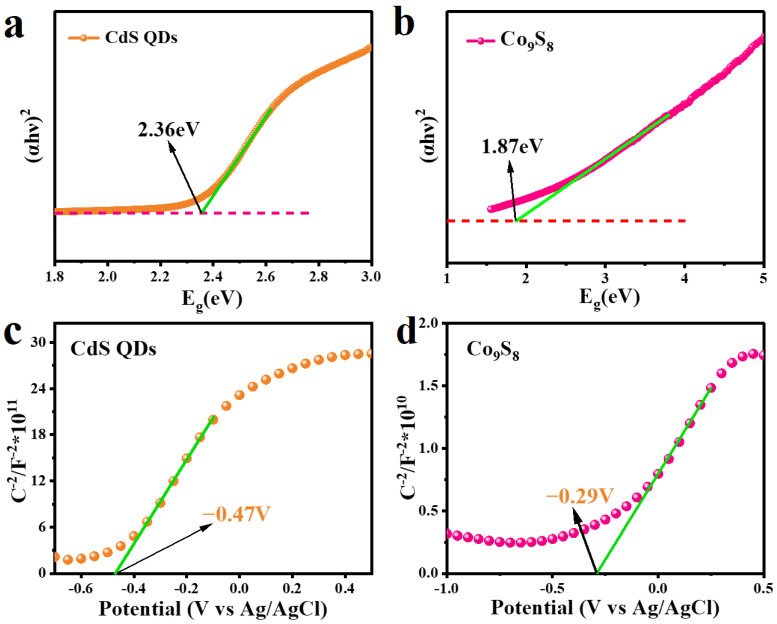
The band gap energy of (**a**) CdS QDs and (**b**) Co_9_S_8_. Mott–Schottky plots of (**c**) CdS QDs and (**d**) Co_9_S_8_.

The band structure information of CdS and Co_9_S_8_ can be acquired through UV–vis absorption spectra (Figure 4b) and Mott–Schottky plots (Figure 8). The band gap energy (E_g_) of the synthesized samples is determined through the Tauc equation: (αhν)^2^ = A(hν − E_g_), where α, ν, h and A are the absorption coefficient, frequency of light, Planck’s constant and proportionality constant, respectively. As depicted in Figure 8a,b, CdS QDs and Co_9_S_8_ exhibit E_g_ of 2.36 eV and 1.87 eV, respectively. The Mott–Schottky (M-S) method is employed to ascertain the semiconductor type and band potential. Figure 8c,d illustrates that both CdS and Co_9_S_8_ display a positive slope, indicating their n-type semiconductor nature [50]. From the M-S diagram, it is evident that the flat band potential (V_fb_) for CdS QDs is −0.47 V, while that of Co_9_S_8_ is −0.29 V (vs. Ag/AgCl). Since the conduction potential (E_CB_) of n-type semiconductors is approximately −0.2 V negative than V_fb_, it can be calculated that the E_CB_ of CdS and Co_9_S_8_ are −0.67 V and −0.49 V (vs. Ag/AgCl), respectively [51]. According to the conversion relationship, we determine that the E_CB_ of CdS is −0.47 V, while that of Co_9_S_8_ is −0.29 V (vs. NHE). Based on the E_g_ of CdS and Co_9_S_8_, their valence band potential (E_VB_) can be calculated as 1.89 V and 1.58 V using the formula E_VB_ = E_CB_ + E_g_.

**Figure 9:**
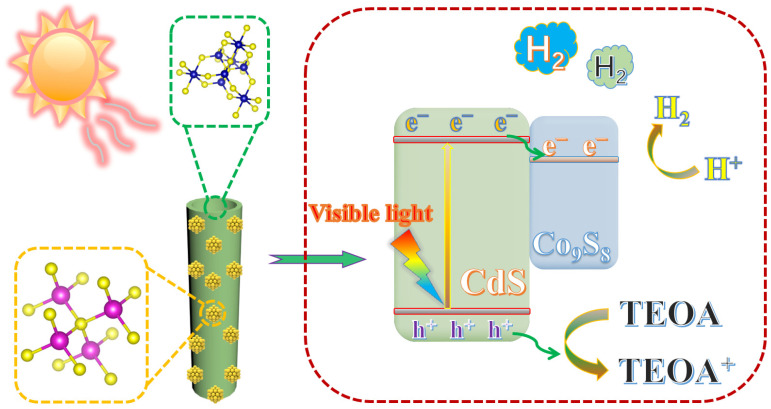
Mechanism diagram of CdS QDs-Co_9_S_8_ in photocatalytic hydrogen production driven by visible light.

Based on the aforementioned characterizations, a potential mechanism for visible-light-driven photocatalytic hydrogen evolution by CdS QDs-Co_9_S_8_ has been put forward. The band positions and band gaps of CdS and Co_9_S_8_ have been determined through Mott–Schottky analysis and UV–vis DRS transformation plots. Since Co_9_S_8_ (−0.29 V) exhibits a lower conduction band potential (E_CB_) than CdS (−0.47 V), it suggests that the photogenerated electrons from CdS will be transferred to the CB of Co_9_S_8_. As exhibited in Figure 9, the irradiation of visible light results in the excited electrons in the valence band (VB) of CdS QDs jumping to the CB, accompanied by the generation of photogenerated holes in the VB. Due to the tight interfacial contact between CdS QDs and Co_9_S_8_, photogenerated electrons are transferred from the CB of CdS QDs to the CB of Co_9_S_8_ instead of being trapped by holes. Subsequently, the electrons that have accumulated on Co_9_S_8_ combine with H^+^ to form H_2_. Meanwhile, the remaining photogenerated holes in the VB of CdS rapidly oxidize the sacrificial agent triethanolamine, forming a complete reaction cycle. Furthermore, the nanotube structure of Co_9_S_8_ provides a multitude of active sites for photocatalytic hydrogen production reactions, and combined with the multiple reflections of light in the hollow structure of Co_9_S_8_, the photocatalytic H_2_ evolution performance of the CdS QDs-Co_9_S_8_ composite is significantly enhanced.

## 3. Experimental Section

### 3.1. Materials

Anhydrous chromium chloride (CdCl_2_), sodium hydroxide (NaOH), cobalt chloride hexahydrate (CoCl_2_·6H_2_O), 3-aminopropyl triethoxysilane (APTES) and 3-mercaptopropionic acid (C_3_H_6_O_2_S, MPA) were supplied by Shanghai Macklin Biochemical Co., Ltd. (Shanghai, China). Triethanolamine (C_6_H_15_NO_3_, TEOA) and anhydrous ethanol (C_2_H_6_O) were purchased from Xilong Scientific Co., Ltd. (Shantou, China). Sodium sulfide 9-hydrate (Na_2_S·9H_2_O) was supplied by Shanghai Aladdin Biochemical Technology Co., Ltd. (Shanghai, China). Urea (CH_4_N_2_O) was purchased from Sinopharm Chemical Reagent Co., Ltd. (Shanghai, China).

### 3.2. Preparation of CdS QDs

In a typical experiment, 1.7 mmol MPA (3-mercaptopropionic acid) and 1 mmol CdCl_2_ were dissolved in 20 mL of deionized water. The pH was then modulated to about 10 through the addition of sodium hydroxide solution. The resulting solution was then diverted into a three-necked flask, which was sealed and the air outlet preserved. Subsequently, 5 mL of Na_2_S solution (0.2 mol/L) was added to the above solution in an atmosphere of argon gas and magnetically stirred. The solution was then heated to 373 K, after which the yellow solution was agitated for 0.5 h. Once the solution had cooled, 50 mL of ethanol was added to precipitate it. The resulting yellowish product was obtained after extraction, filtration, washing and drying.

### 3.3. Preparation of Co_9_S_8_ Nanotubes

The preparation process of Co_9_S_8_ nanotubes referred to the two-step hydrothermal method in previous work [52,53]. Firstly, Co(CO_3_)_0.35_Cl_0.20_(OH)_1.10_ nanorods were synthesized as a precursor for Co_9_S_8_ nanotubes. This was achieved by dissolving CoCl_2_·6H_2_O (5 mmol) and CH_4_N_2_O (5 mmol) in 40 mL deionized water and ultrasounding the solution for 30 min. Subsequently, the solution was diverted into a 50 mL Teflon autoclave and reacted in a 393 K oven for 10 h. The precipitate was then gathered through centrifugation and washed multiple times with anhydrous ethanol and deionized water. The pink precursor was obtained following drying at 333 K for several hours. Subsequently, the synthesized Co(CO_3_)_0.35_Cl_0.20_(OH)_1.10_ precursors (110 mg) were mixed to 40 mL of Na_2_S solution (5 mg/mL) in the Teflon liner and stirred for an hour. The liner was then diverted into a stainless-steel autoclave and heated to a temperature of 433 K for a period of 8 h. During the vulcanization process, the inner material of the rod-like precursor underwent a reaction and fell off, thereby obtaining the Co_9_S_8_ of the hollow nanotube structure. Subsequently, the product was isolated through suction filtration, washed with anhydrous ethanol and deionized water and dried at 333 K for 12 h, and the dried product (black powder) was collected for further processing.

### 3.4. Positive Electrochemical Treatment of Co_9_S_8_ Nanotubes

The prepared 100 mg Co_9_S_8_ nanotubes were dispersed in 50 mL C_2_H_5_OH and ultrasonic until the solution was uniform. Then, 2 mL of APTES (3-aminopropyl triethoxysilane) solution was added to the ultrasonic-treated Co_9_S_8_ nanotube ethanol solution and stirred for 20 min. Subsequently, the product was maintained in a water bath at 333 K for a period of four hours, centrifuged and washed with anhydrous ethanol and deionized water on several occasions. The obtained product was then dried in a 333 K oven and collected for use.

### 3.5. Electrostatic Assembly of CdS QDs-Co_9_S_8_

Typically, 50 mg CdS QDs was dispersed in 50 mL deionized water and ultrasounded for 5 min. A certain proportion of 5%/10%/30% (2.5 mg/5 mg/15 mg) electropositive Co_9_S_8_ nanotubes were dispersed in deionized water by the same method described above and ultrasonic. After ultrasound, the Co_9_S_8_ nanotube solution was injected into the CdS QDs solution and stirred for a period of 2.5 h. Subsequently, the mixed solution was subjected to centrifugation and multiple washes with deionized water, after which it was dried in an oven at 333 K to yield the dried yellowish-green product.

### 3.6. Activity Evaluation of Photocatalytic H_2_ Evolution 

Photocatalytic H_2_ production was conducted within a 50 mL closed quartz reactor. Typically, 1 mL of triethanolamine (TEOA) and 5 mL of deionized water were added to a sealed quartz reactor containing 5 mg of CdS QDs-Co_9_S_8_ composite photocatalyst, followed by ultrasound until the solution was uniform. Subsequently, pure argon gas was implanted into the quartz reactor for half an hour to remove residuary air. A 300 W xenon lamp (PLS-SXE300D, Perfectlight, Beijing, China) with an ultraviolet cut-off filter (λ ≥ 420 nm) was used as the light source. Following a two-hour illumination period, 1 mL of mixed gas was injected into the gas chromatograph (GC7900, Techcomp, Shanghai, China) to detect the peak areas of hydrogen and argon, and the hydrogen production rate of the photocatalyst was then converted according to the hydrogen production coefficient given. Additionally, the stability of the CdS QDs-Co_9_S_8_ composite photocatalyst was evaluated by conducting tests for 5 cycles under the same conditions after centrifugation, washing and drying.

## 4. Conclusions

In summary, Co_9_S_8_ hollow nanotubes were prepared through a two-step hydrothermal approach as a cocatalyst, and the CdS QDs-Co_9_S_8_ composite photocatalysts were successfully prepared through a straightforward electrostatic self-assembly method. The electrostatic self-assembly strategy ensures a tight interfacial contact between CdS QDs and Co_9_S_8_ nanotubes. By adjusting the proportion of Co_9_S_8_ nanotubes in the composite, the photocatalytic hydrogen evolution rate of the optimal CdS QDs-30%Co_9_S_8_ nanotubes is 9642.7 μmol·g^−1^·h^−1^, approximately 60.3 times that of blank CdS QDs. The cyclic experiment demonstrates that the introduction of Co_9_S_8_ cocatalysts effectively prevents photocorrosion on the surface of CdS QDs. A series of characterization experiments illustrate that the introduction of Co_9_S_8_ hollow nanotubes resulted in a more uniform and dispersed growth of CdS QDs particles, as well as the promotion of the separation and migration of photogenerated carriers. As a result, the CdS QDs-Co_9_S_8_ composite exhibits excellent activity and stability in photocatalytic hydrogen production. This work provides new perspectives for the rational construction of stable, environmentally friendly and highly active composite photocatalysts to realize efficient photocatalytic H_2_ evolution.

## Figures and Tables

**Table 1:** Contrast of the H_2_ production performance of the CdS-based photocatalysts.

Photocatalysts	Light Sources	Sacrificial Agents	H_2_ (μmol·g^−1^·h^−1^)	Reference
CdS QDs-30% Co_9_S_8_	300 W Xe lamp (λ ≥ 420 nm)	TEOA	9642.7	this work
CdS/TiO_2_@Ti_3_C_2_	300 W Xe lamp (λ ≥ 420 nm)	TEOA	3115.0	[30]
CdS QDs/Ni_2_P/B-TiO_2_	300 W Xe arc lamp	Na_2_S/Na_2_SO_3_	3303.9	[31]
CdS/Au/KTaO_3_	Xe lamp (λ ≥ 420 nm)	Na_2_S/Na_2_SO_3_	2892.0	[32]
CdS QDs/CeO_2_	300 W Xe lamp (λ ≥ 300 nm)	Na_2_S/Na_2_SO_3_	101.1	[33]
Ni@NiO/CdS	500 W Xe lamp	TEOA	4380.0	[34]
CuS/CdS	300 W Xe lamp (λ ≥ 420 nm)	lactic acid (10 vol%)	5617.0	[35]
UiO-66-NH_2_@CdS	300 W Xe lamp (λ ≥ 420 nm)	Na_2_S/Na_2_SO_3_	2028.5	[36]
ZnO-Cu-CdS	300 W Xe lamp (λ ≥ 420 nm)	glycerol	4655.0	[37]
Ag_2_S-CdS	300 W Xe lamp (λ ≥ 420 nm)	lactic acids (2 vol%)	777.3	[38]

## Data Availability

Data are contained within the article.

## Supplementary Materials

The following supporting information can be downloaded at: https://www.mdpi.com/article/10.3390/molecules29153530/s1. Figure S1. Schematic representation of the samples for (a) CdS QDs, (b) Co_9_S_8_ and (c) CdS QDs-Co_9_S_8._ Figure S2. (a) TEM image and (b) HRTEM image of Co_9_S_8._ Figure S3. Pore size distributions of (a) CdS QDs, (b) Co_9_S_8_ and (c) CdS QDs-30%Co_9_S_8._ Figure S4. Nitrogen adsorption–desorption isotherms of (a) CdS QDs-5%Co_9_S_8_, (b) CdS QDs-10%Co_9_S_8_ and (c) CdS QDs-50%Co_9_S_8._ Table S1. Summary of the ICP analysis results of the samples of CdS QDs-5%Co_9_S_8_, CdS QDs-10%Co_9_S_8_, CdS QDs-30%Co_9_S_8_ and CdS QDs-50%Co_9_S_8._ Table S2. The average pore size distributions of the prepared photocatalysts.
