# Supplementary material for: Electrostatic Self-Assembly of CdS Quantum Dots with Co9S8 Hollow Nanotubes for Enhanced Visible Light Photocatalytic H2 Production
Source: Molecules. 2024 Jul 26;29(15):3530. doi: 10.3390/molecules29153530 (PMC11314185; doi:10.3390/molecules29153530)
Supplement: Supplementary file 1 [file molecules-29-03530-s001.zip › molecules-3092844-supplementary.pdf]

Supporting information

Electrostatic Self-Assembly of CdS Quantum Dots with Co_9S_8 Hollow Nanotubes for Enhanced Visible Light Photocatalytic H_2 Production

Yuqing Yan [1], Yonghui Wu [1], Chenggen Lu [1], Yu Wei [1], Jun Wang [1], Bo Weng [2], Wei-Ya Huang [1], Jia-Lin Zhang [1], Kai Yang [1] and Kangqiang Lu [1,*]

[1] Jiangxi Provincial Key Laboratory of Functional Crystalline Materials Chemistry, School of Chemistry and Chemical Engineering, Jiangxi University of Science and Technology, Ganzhou 341000, China; 18797912889@163.com (Y.-Q.Y.); 13207070350@163.com (Y.-H.W.); 17779524039@163.com (C.-G.L.); 18225906201@163.com (Y.W.); wj15270473375@126.com (J.W.); hweiya@126.com (W.-Y.H.); jialinz2007@163.com (J.-L.Z.); yangkai@jxust.edu.cn (K.Y.)

[2] CAS Key Laboratory of Urban Pollutant Conversion, Institute of Urban Environment, Chinese Academy of Sciences, Xiamen 361021, China; bweng@iue.ac.cn

* Correspondence: kqlu@jxust.edu.cn

1.1 Characterization methods

The crystal phase structure and composition of the catalyst were determined by X-ray powder diffraction (Bruker D8 Advance, Billerica, MA, USA). Scanning electron microscope (FESEM Zeiss Sigma 500, Oberkochen, Batenwerburg, Germany)) and transmission electron microscope (TEM, JEM-2100F, Jeol, Akishima, Tokyo) were used to analyze the morphology and microstructure of the composite photocatalyst. The elemental composition of the composite photocatalyst was measured by inductively coupled plasma emission spectrometer (ICP-OES). The composition and valence of the composite photocatalyst were analyzed by Thermo Fisher K-Alpha Plus (X-ray photoelectron spectroscopy). The UV-visible diffuse reflectance spectrometer (DRS, Shimadzu UV-2600, Kyoto, Japan) was utilized to test the optical response of the catalyst. Photoluminescence (PL) spectra were obtained using a spectrofluorometer (FLS 980, Edinburgh Instruments Ltd., Edinburgh, UK) with an excitation wavelength of 500 nm. The Zeta potential (ζ) in deionized water was determined by dynamic light scattering analysis (Zetasizer Nano ZS90, Malvern, UK) at room temperature. Furthermore, all the electrochemical measurements of the photocurrent and the electrochemical impedance spectra (EIS) were carried out in the three-electrode cell, in which Ag/AgCl was used as a reference electrode, a Pt wire was used as a counter electrode, and an indium in oxide (ITO) conductive glass was used with the samples as a working electrode in 0.1 M Na_2SO_4 electrolyte (pH=7.56), all measurements were carried out on CH instruments CHI-660E electrochemical workstation (Shanghai Chenhua CHI-660E, Shanghai, China). The specific surface area and pore size of the composite photocatalyst were determined by nitrogen physical adsorption desorption (ASAP2020).

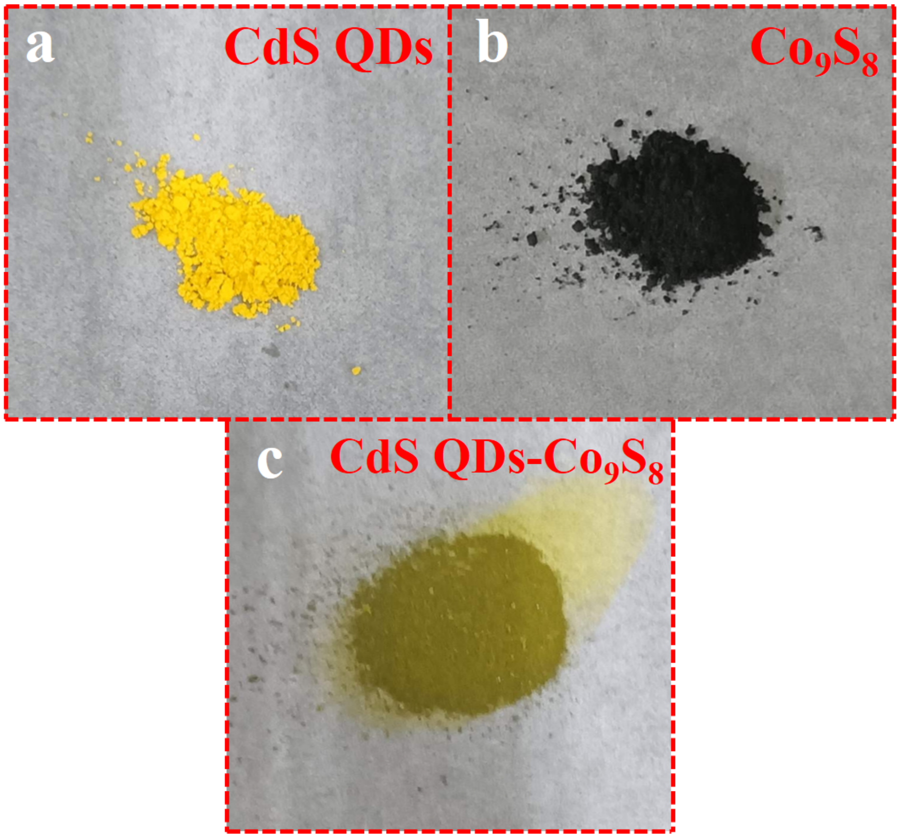

Figure S1. Schematic representation of the samples for (a) CdS QDs, (b) Co_9S_8 and (c) CdS QDs-Co_9S_8.

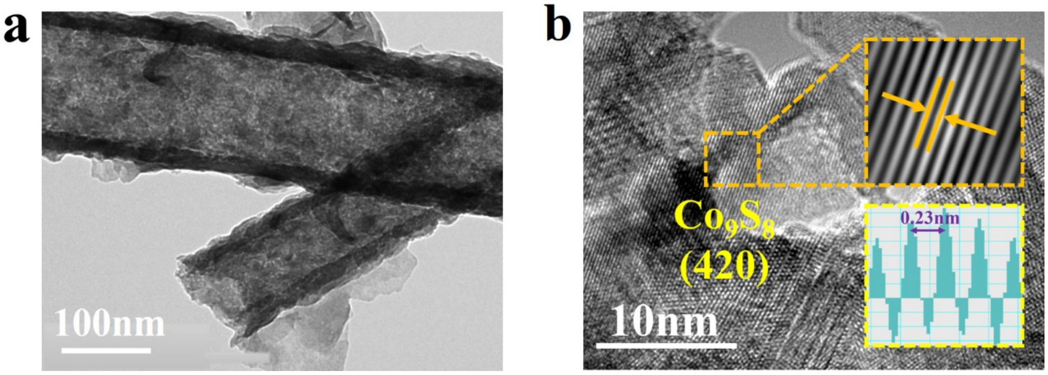

Figure S2. (a) TEM image and (b) HRTEM image of Co_9S_8.

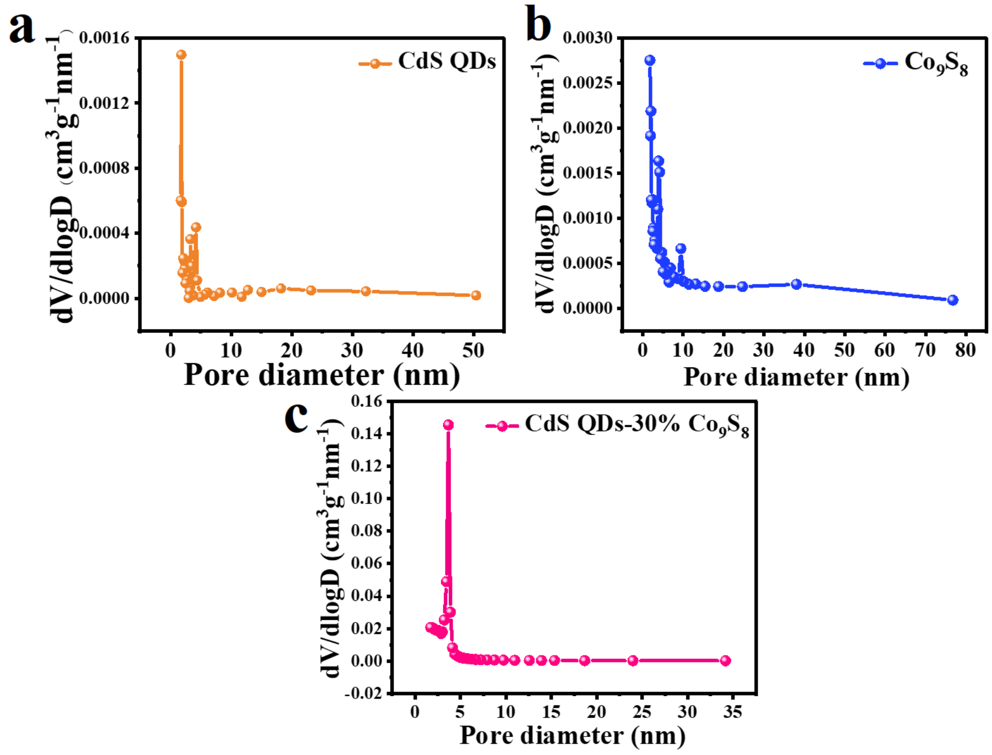

Figure S3. Pore size distributions of (a) CdS QDs, (b) Co_9S_8 and (c) CdS QDs-30%Co_9S_8.

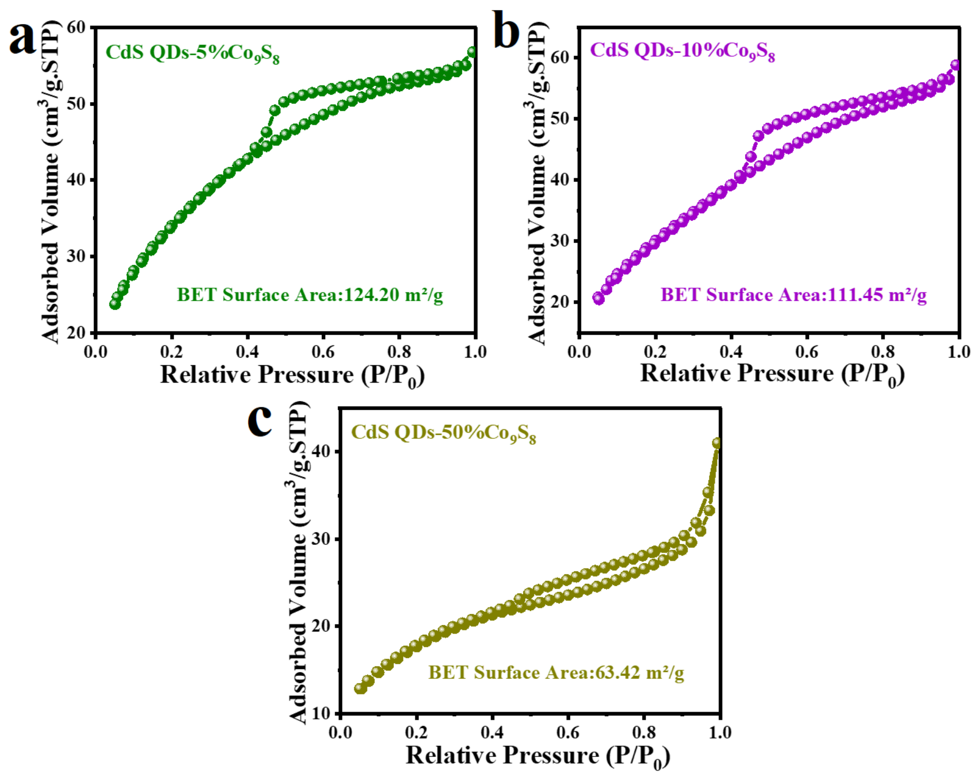

Figure S4. Nitrogen adsorption–desorption isotherms of (a) CdS QDs-5%Co_9S_8, (b) CdS QDs-10%Co_9S_8 and (c) CdS QDs-50%Co_9S_8.

Table S1

Summary of the ICP analysis results of the samples of CdS QDs-5%Co_9S_8, CdS QDs-10%Co_9S_8, CdS QDs-30%Co_9S_8 and CdS QDs-50%Co_9S_8.

Samples	Cd (ppm)	Co (ppm)	S (ppm)
CdS QDs-5%Co_9S_8	60.5	1.4	13.0
CdS QDs-10%Co_9S_8	58.6	2.9	13.7
CdS QDs-30%Co_9S_8	54.6	8.2	15.8
CdS QDs-50%Co_9S_8	41.0	15.7	12.8

Table S2

The average pore size distributions of the prepared photocatalysts.

Photocatalysts	CdS QDs	Co_9S_8	CdS QDs-30% Co_9S_8
Average pore size (nm)	15.58	15.15	3.62
